# Targeting Cellular Calcium Homeostasis to Prevent Cytokine-Mediated Beta Cell Death

**DOI:** 10.1038/s41598-017-05935-4

**Published:** 2017-07-17

**Authors:** Amy L. Clark, Kohsuke Kanekura, Zeno Lavagnino, Larry D. Spears, Damien Abreu, Jana Mahadevan, Takuya Yagi, Clay F. Semenkovich, David W. Piston, Fumihiko Urano

**Affiliations:** 1 Department of Pediatrics, Washington University School of Medicine, St. Louis, MO 63110 USA; 2 Department of Molecular Pathology, Tokyo Medical University, Tokyo, 160-8402 Japan; 3 Department of Cell Biology and Physiology, Washington University School of Medicine, St. Louis, MO 63110 USA; 4 Department of Medicine, Division of Endocrinology, Metabolism, and Lipid Research, Washington University School of Medicine, St. Louis, MO 63110 USA; 5 Department of Pathology and Immunology, Washington University School of Medicine, St. Louis, MO 63110 USA

## Abstract

Pro-inflammatory cytokines are important mediators of islet inflammation, leading to beta cell death in type 1 diabetes. Although alterations in both endoplasmic reticulum (ER) and cytosolic free calcium levels are known to play a role in cytokine-mediated beta cell death, there are currently no treatments targeting cellular calcium homeostasis to combat type 1 diabetes. Here we show that modulation of cellular calcium homeostasis can mitigate cytokine- and ER stress-mediated beta cell death. The calcium modulating compounds, dantrolene and sitagliptin, both prevent cytokine and ER stress-induced activation of the pro-apoptotic calcium-dependent enzyme, calpain, and partly suppress beta cell death in INS1E cells and human primary islets. These agents are also able to restore cytokine-mediated suppression of functional ER calcium release. In addition, sitagliptin preserves function of the ER calcium pump, sarco-endoplasmic reticulum Ca^2+^-ATPase (SERCA), and decreases levels of the pro-apoptotic protein thioredoxin-interacting protein (TXNIP). Supporting the role of TXNIP in cytokine-mediated cell death, knock down of TXNIP in INS1-E cells prevents cytokine-mediated beta cell death. Our findings demonstrate that modulation of dynamic cellular calcium homeostasis and TXNIP suppression present viable pharmacologic targets to prevent cytokine-mediated beta cell loss in diabetes.

## Introduction

Type 1 diabetes mellitus (T1DM) results from an autoimmune attack on insulin producing beta cells that leads to immune cell infiltration of the pancreatic islets, inflammation, and beta cell death. Several studies have employed immunosuppression to prevent T1DM, but this modality alone does not alter the course of T1DM in humans^1–4^. This is likely secondary to the fact that there are some intrinsic features in the propagation of islet inflammation and beta cell death in T1DM that persist despite immunosuppression. Our goal is to target beta cell specific molecular pathways involved in initiation of autoimmunity and progression of cytokine-mediated beta cell death, which may identify novel therapies for beta cell preservation in T1DM. ER stress has been implicated in development of autoimmunity, propagation of insulitis and beta cell death in T1DM^5–13^. As ER stress can potentially be involved in diabetes from development of autoimmunity to beta cell death, it is an attractive target for preventing beta cell death in T1DM.

Cytokines are potent inducers of ER stress and are known to promote autoimmune destruction of islets in T1DM^10, 13–17^. Cytokine stress leads to generalized ER dysfunction and altered cellular calcium homeostasis prior to initiating cell death. Specifically, cytokine exposure leads to pathogenic alterations in intracellular free calcium levels, including ER calcium depletion and cytosolic calcium elevation in beta cells^10, 18–20^. In addition to coordinating protein synthesis and folding, the ER is involved in calcium storage and signaling, and is the source of both pro and anti-apoptotic signaling pathways^21, 22^. A high level of ER calcium is required for proper ER function in the context of protein folding and participation in cell signaling cascades. We have recently shown that ER calcium depletion, followed by a subsequent increase in cytoplasmic calcium, is seen in beta cells treated with inflammatory cytokines^20^. Thus, targeting ER and cellular calcium homeostasis may prevent cytokine-mediated beta cell death in T1DM. Here we report that two well-characterized small molecules, dantrolene and sitagliptin, preserve functional ER calcium release in beta cells treated with inflammatory cytokines and suppress beta cell death.

## Results

**Figure 1:**
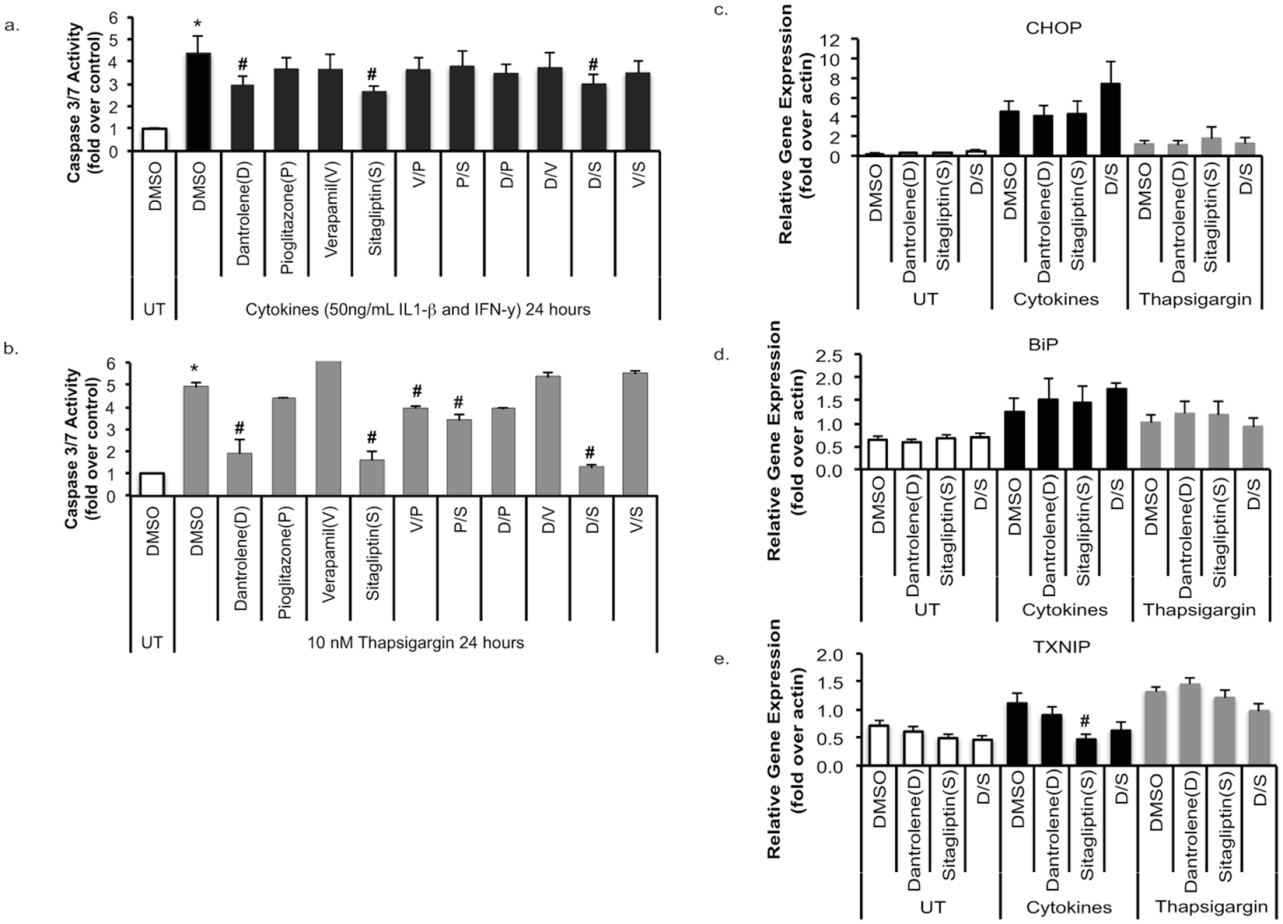
Dantrolene and Sitagliptin Protect INS-1E Cells From Cytokine and ER Stress Induced Cell Death. (**a**,**b**) INS-1E cells were pre-treated with 100 nM dantrolene, 10 μM pioglitazone, 10 μM verapamil, or 200 nM sitagliptin for 24 hours then stressed for 24 hours with cytokine cocktail (IL-1β and IFN-γ 50 ng/mL) or thapsigargin 10 nM. Apoptotic cell death was measured via caspase 3/7 activity assay. (**c**–**e**) The ER stress markers CHOP BiP and TXNIP were measured using quantitative RT-PCR. Data are expressed as mean ± SEM from at least three independent experiments. ^#^p < 0.05 compared to cytokine or TG treated cells via unpaired t-test.

To determine if modulation of ER and cytoplasmic free calcium levels can protect beta cells from cytokine-mediated cell death, we pretreated rat INS1-E cells with well-characterized Food and Drug Administration approved agents known to modulate cellular calcium levels. Drugs known to target cytosolic calcium levels included verapamil and sitagliptin. Drugs known to target ER calcium levels included pioglitazone and dantrolene^16, 23–25^. To determine if there would be any additive effect by altering both ER and cytosolic calcium levels, drugs were studied individually as well as in combination with one another. Cells were then challenged with a cytokine cocktail or the ER stress inducing agent thapsigargin. As expected, cytokines and thapsigargin significantly increased cell death as indicated by increased caspase 3/7 activity levels (Fig. 1a and b). In both cytokine and thapsigargin treated cells, dantrolene and sitagliptin significantly decreased beta cell death (Fig. 1a and b).

As dantrolene and sitagliptin provided significant protection against cytokine and ER stress-induced beta cell apoptosis, further studies were done to determine effects on ER stress, intracellular calcium levels and pro-apoptotic signals. Dantrolene and sitagliptin did not prevent cytokine-induced expression of the canonical ER stress markers CHOP or BiP^26, 27^ (Fig. 1c and d). However, sitagliptin did significantly lower expression of the pro-apoptotic thioredoxin-interacting protein (TXNIP) (Fig. 1e).

**Figure 2:**
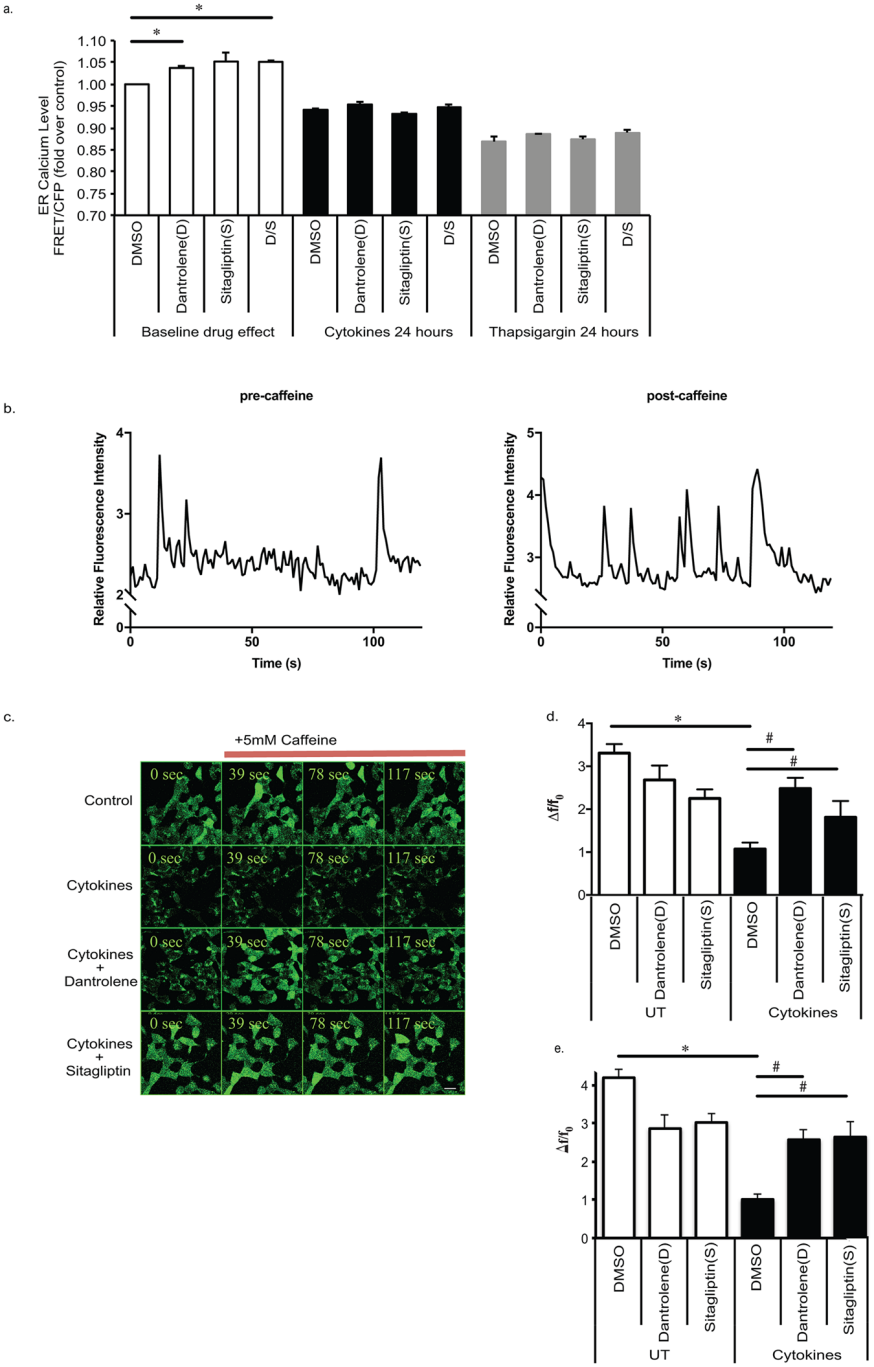
Dantrolene and Sitagliptin Restore Caffeine Mediated ER Calcium Release in Cytokine and ER Stressed Βeta Cells. (**a**) INS-1E cells were treated with 100 nM dantrolene, 200 nM sitagliptin or a combination of the two drugs for 24 hours followed by treatment with 10 nM thapsigargin for 24 hours or 6–24 hours of cytokine cocktail (IL-1β and IFN-γ 50 ng/mL) treatment. ER calcium levels were monitored in INS-1E cells stably expressing the D1ER probe via FACS analysis and presented as FRET/CFP. Fluorescence was monitored continuously for two minutes in INS-1E cells loaded with Fluo-4 AM imaged at baseline and after 5 mM caffeine stimulation and single cell calcium oscillation tracings were produced as depicted in panel b. Panel c shows representative single plane images at different times during the 2 minute acquisition after the addition of caffeine. Graphical representation of the relative change in fluorescence intensity averaged over 2 minutes of image acquisition, expressed as ΔF/F_0_ (ΔF is the change in fluorescence intensity of the caffeine stimulated cells relative to the basal condition F_0_) is displayed in the presence (panel d) and absence of calcium in the media (panel e). The average represents values over 2 minutes in 50 cells normalized for variations in the concentration of the dye in different cells. Data are expressed as mean ± SEM from at least three independent experiments. *p < 0.05 compared to control; ^#^p < 0.05 compared to cytokine or TG treated cells via one way ANOVA test.

To determine if dantrolene and sitagliptin had significant effects on cytokine-induced ER calcium depletion, separate studies were done to monitor both baseline ER calcium levels as well as functional ER calcium release. To monitor baseline ER calcium levels, we created an INS-1E cell line stably expressing the ER-localized calcium sensor D1ER^20, 28^. Experiments in INS-1E-D1ER cells show that dantrolene and the combination of dantrolene and sitagliptin significantly increased ER calcium levels at baseline (Fig. 2a). However, despite increasing ER calcium at baseline, the drugs were not able to protect INS-1E cells from cytokine or thapsigargin-induced ER calcium depletion (Fig. 2a).

In order to examine drug effects on ER’s function to release calcium, caffeine-stimulated ER calcium release was monitored over time using confocal microscopy. Caffeine has been used previously to induce ER calcium release via activation of ryanodine receptors^17, 29^. The administration of caffeine, overall, resulted in increased frequency and magnitude of cytosolic calcium oscillations, as shown by the representative traces (Fig. 2b). This effect of caffeine can be seen for several minutes in healthy beta cells. Thus, monitoring caffeine-mediated calcium oscillations gives an indirect measure of the dynamic activity of the ER over time. To monitor ER calcium release, INS-1E cells loaded with the calcium indicator dye Fluo-4 were pre-treated with vehicle, dantrolene or sitagliptin, and exposed to a cytokine cocktail. The drugs were washed out prior to imaging, and Fluo-4 signal was imaged in the cells at baseline and after stimulation with caffeine. The value ΔF/F0 represents the change of Fluo-4 fluorescence when caffeine is added to the cells (ΔF) normalized to its initial signal (F0) as measured and averaged over a 2 minute period. As expected, control cells exhibited a robust increase in Fluo-4 signal upon caffeine exposure (Fig. 2c and d). Treatment with cytokines prevented ER calcium release so that there was no relative change in Fluo-4 intensity upon caffeine stimulation (Fig. 2c and d). In contrast, dantrolene and sitagliptin treatment resulted in a significant restoration of caffeine-mediated calcium release in cytokine treated cells. Experiments performed in calcium free buffer resulted in similar findings (Fig. 2e), which indicate that extracelluar calcium is not affecting these results. Taken together, the data suggest that while baseline ER calcium levles remained depleted dantrolene and sitagliptin were able to restore functional ER calcium release in cytokine-stressed beta cells.

**Figure 3:**
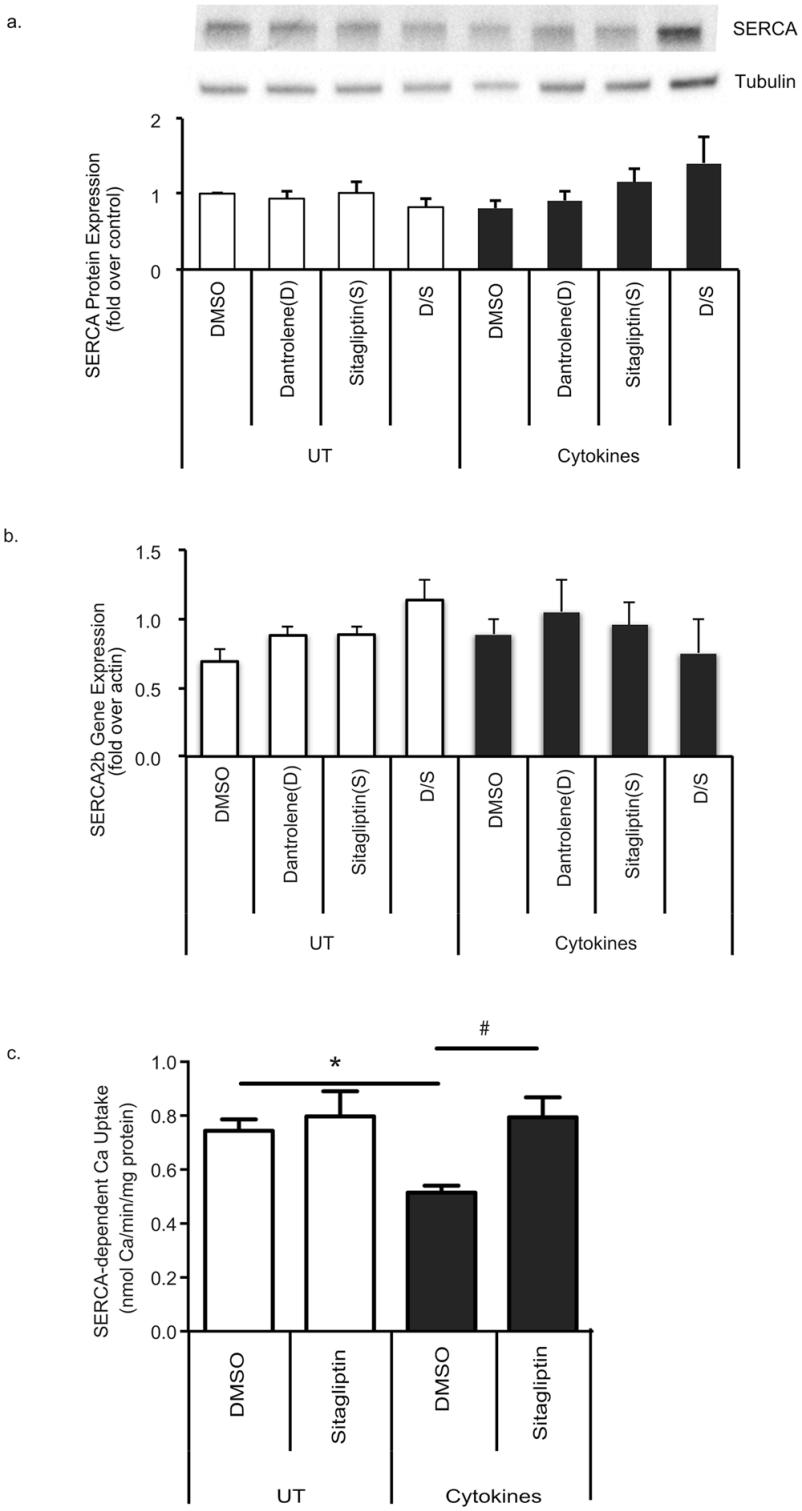
Sitagliptin Maintains SERCA Activity During Cytokine Stress. INS-1E cells were pre-treated with 100 nM dantrolene or 200 nM sitagliptin for 24 hours then stressed for 24 hours with cytokine cocktail (IL-1β and IFN-γ 50 ng/mL). (**a**) SERCA protein levels were measured by immunoblot. Full-length blot can be seen on-line (Supplementary Figure 1). (**b**) SERCA2b mRNA levels were measured by real-time PCR. (**c**) SERCA activity levels were monitored and expressed as nmol Ca/min/mg protein. Data are expressed as mean ± SEM from at least three independent experiments. *p < 0.05 compared to control; ^#^p < 0.05 compared to cytokine or TG treated cells via one way ANOVA test.

The expression of the sarco-endoplasmic reticulum Ca^2+^-ATPase (SERCA) calcium transporter has been shown to be decreased by cytokine treatment^10^. Thus, we sought to determine if the drugs’ effect on functional ER calcium release was related to modulation of levels of SERCA. Immunoblotting and real-time PCR analysis did not indicate that dantrolene or sitagliptin effected SERCA2b mRNA or protein levels of SERCA in INS-1E cells at baseline (Fig. 3a and b). In addition neither drug had a significant effect on SERCA levels after cytokine stress (Fig. 3a and b). Next, we looked at SERCA activity during cytokine stress. SERCA activity was significantly decreased with cytokine treatment and restored by sitagliptin treatment (Fig. 3c). Collectively, these results suggest sitagliptin does not increase SERCA levels but does lead to preservation of SERCA activity during cytokine stress.

**Figure 4:**
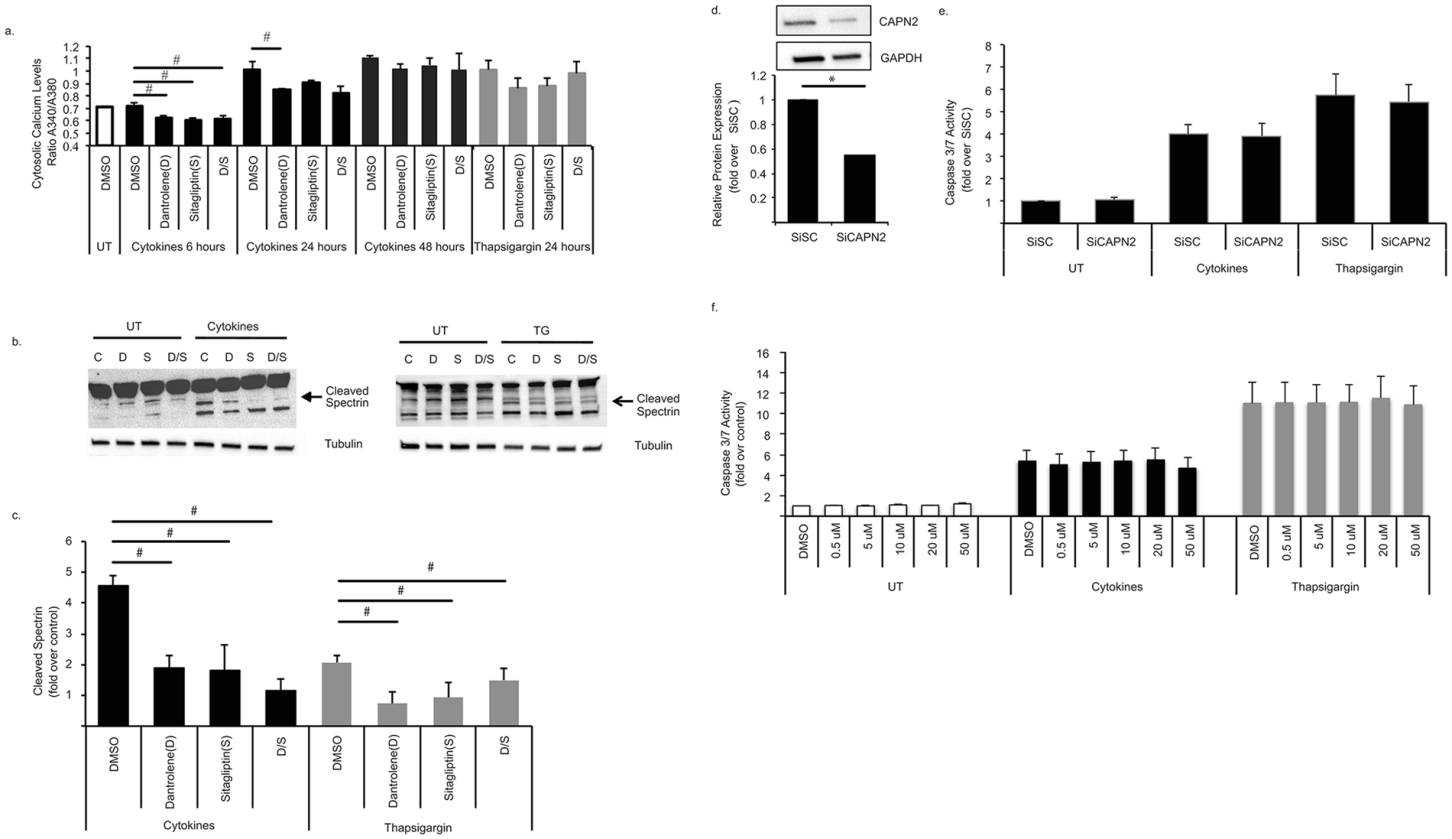
Dantrolene and Sitagliptin Significantly Decrease Calpain Activation in INS-1E Cells. (**a**) INS-1E cells were pre-treated with 100 nM dantrolene or 200 nM sitagliptin for 24 hours then stressed for 6–48 hours with cytokine cocktail (IL-1β and IFN-γ 50 ng/mL) or 10 nM thapsigargin. (**a**) cytosolic free calcium activity was determined in INS-1E cells via Fura-2 assay and presented as A340/A380. (**b**) Calpain activation was measured via immunoblot for spectrin cleavage products and (**c**) quantitative densitometry was performed via ImageJ software (NIH). Full-length blot can be seen on-line (Supplementary Figure 1). (**d**) INS-1E cells were transfected with SiRNA against a scrambled construct (SiSC) or calpain 2 (SiCAPN2) and immunoblot was performed for calpain-2 expression. (**e**) SiSC and SiCAPN2 transfected INS-1E cells were then exposed to cytokines (IL-1β and IFN-γ 50 ng/mL) or 10 nM thapsigargin for 24 hours and cell death was measured via caspase 3/7 activity. (**f**) INS-1E cellspre-treated for 24 hours with varying doses of calpain inhibitor III. Cells werethen stressed with cytokines (IL-1β and IFN-γ 50 ng/mL) or 10 nM thapsigargin for 24 hours and cell death was monitored via caspase 3/7 activity. Data are expressed asmean ± SEM from at least three independent experiments. ^#^p < 0.05 compared tocytokine or TG treated cells unpaired t-test. Full-length blot can be seen on-line (Supplementary Figure 1).

We next used Fura-2 AM to study the effect of dantrolene and sitagliptin on cytokine and ER stress-induced elevation of cytosolic calcium. After 6 hours of exposure to cytokine cocktail, dantrolene and sitagliptin significantly decreased cytosolic calcium levels compared to cells treated with cytokines alone. However, at 24 hours of cytokine treatment when cytosolic free calcium levels were significantly elevated, only dantrolene significantly lowered cytosolic free calcium levels (Fig. 4a). After 48 hours this effect was lost and neither drug effected cytosolic free calcium levels after thapsigargin treatment (Fig. 4a). These results suggest that the drugs have no significant effect on cytokine-mediated cytosolic calcium elevation.

To further determine the effects of dantrolene and sitagliptin on downstream calcium mediated cell death pathways, we assessed calpain activation. Calpain is a calcium-dependent enzyme whose hyperactivation has been shown to contribute to cell death in various diseases^30, 31^. To measure calpain activity we measured levels of cleaved spectrin, an established calpain cleavage substrate^32^. Both dantrolene and sitagliptin significantly decreased cleaved spectrin levels in response to thapsigargin and cytokine stress (Fig. 4b and c). It has been previously shown that calpain-2 is involved in ER stress-mediated cell death^33, 34^. Thus, we further studied the role of calpain-2 in cytokine-mediated beta cell death using siRNA directed against calpain-2 along with the calpain-1 and calpain-2 inhibiting compound calpain inhibitor III in INS-1E cells. We found that neither siRNA knockdown of calapin-2 nor calpain inhibition suppressed cytokine-mediated cell death in INS-1E cells, suggesting that inactivation of calpain-1 or calpain-2 alone is not sufficient to prevent cytokine and ER stress-mediated beta cell death (Fig. 4d,e and f).

**Figure 5:**
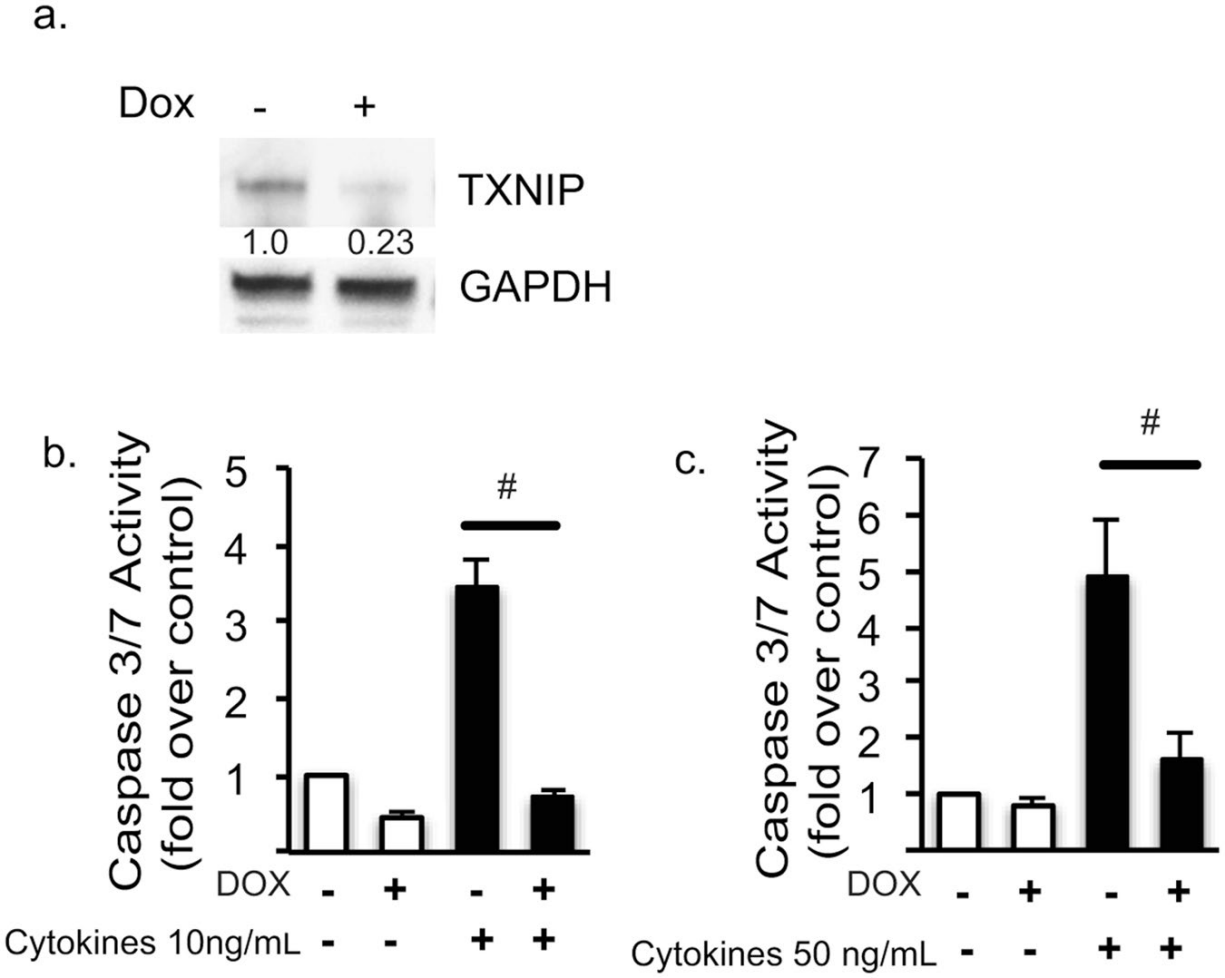
Knock down of TXNIP in INS-1E Cells Prevents Cytokine Induced Caspase 3/7 Activity. (**a**) Western blot showing that TXNIP expression is decreased in tet-shTXNIP cells. Fold over control data are pooled from 5 experiments. Full-length blot can be seen on-line (Supplementary Figure 1). (**b**) Stable Tet-shTXNIP INS1 cell lines were treated with DMSO or doxycycline for 48 hours followed by exposure to a cytokine cocktail of either 10 ng/mL (panel b) or 50 ng/mL (panel c) IL-1β and IFN-γ for 24 hours. Caspase 3/7 activity was measured and data was presented as fold over control for each condition. Statistical analysis was done via two-tailed t tests ^#^P < 0.05, n = 3–8.

TXNIP is up-regulated by ER stress and ER calcium depletion and plays a critical role in beta cell death in cell and mouse models of T1DM^35–37^. Our data show that TXNIP mRNA levels are lower in sitagliptin treated cells stressed with cytokines (Fig. 1e). This raised the possibility that sitagliptin prevents cytokine mediated beta cell death by preventing TXNIP up-regulation. To test this idea, we established inducible TXNIP-knockdown INS1 cells and challenged these cells with inflammatory cytokines (Fig. 5a)^36^. As predicted, suppression of TXNIP protected INS1 cells from cytokine-mediated beta cell death (Fig. 5b and c). These results provide compelling evidence that TXNIP may be a key regulator of cytokine mediated beta cell death. In addition, suppression of TXNIP levels may be a major mechanism by which sitagliptin protects against cytokine mediated beta cell death.

**Figure 6:**
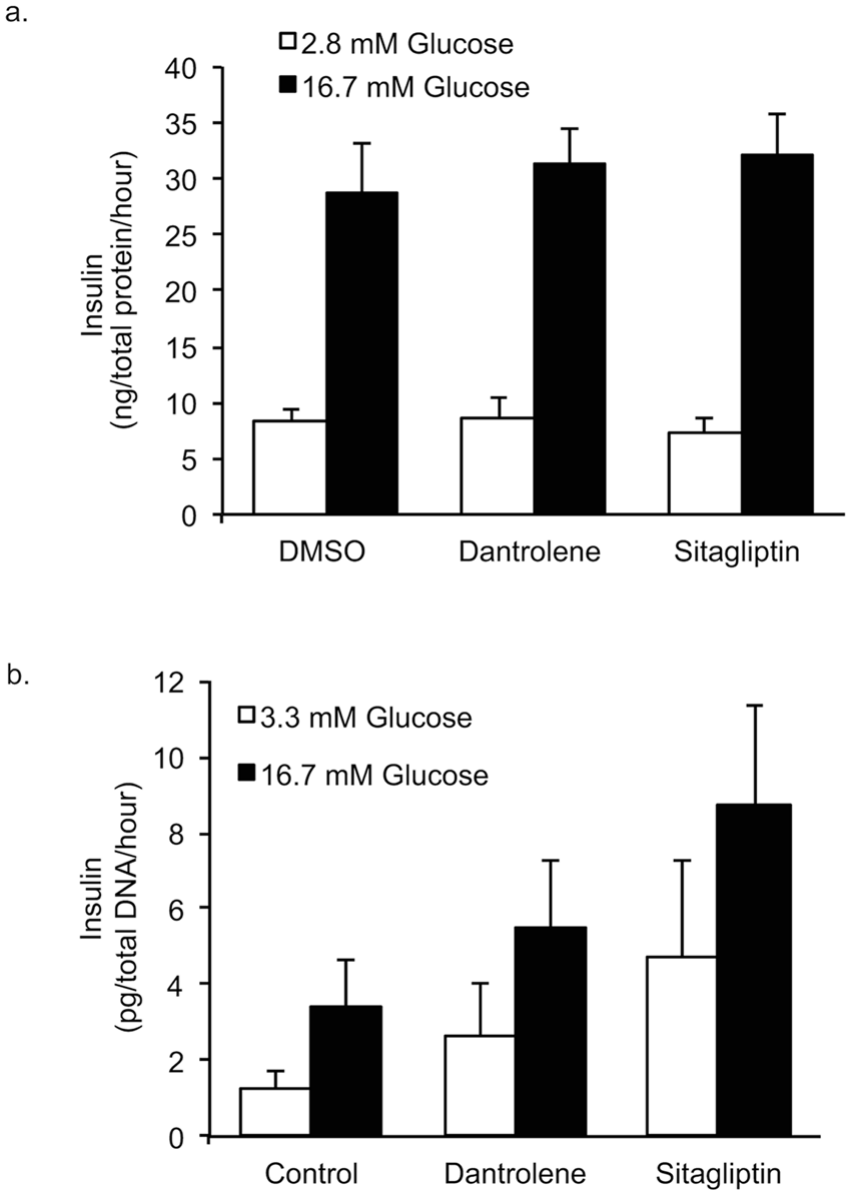
Dantrolene and Sitagliptin do not effect glucose stimulated insulin secretion (GSIS) in INS-1E cells or human islets. (**a**) INS-1E cells were treated with 100 nM dantrolene or 200 nM sitagliptin for 24 hours and GSIS assay was performed. Results are expressed as ng insulin secreted normalized for total protein. (**b**) Human islets from 3 separate donors were treated for 24 hours with 10 μM dantrolene or 200 nM sitagliptin and GSIS assay was performed. Results are expressed as ρg insulin secreted normalized to total DNA. Data are expressed as mean ± SEM. Statistical analysis to determine if drugs had an effect on insulin secretion was done via one-way ANOVA, n = 3–4.

As cellular calcium signaling is key for glucose-stimulated insulin secretion (GSIS), we next set out to determine if the compounds had any effect on GSIS. The effect of dantrolene and sitagliptin on GSIS was examined in INS-1E cells as well as human islets (Fig. 6a and b). Results indicated that treatment with dantrolene and sitagliptin do not affect GSIS.

**Figure 7:**
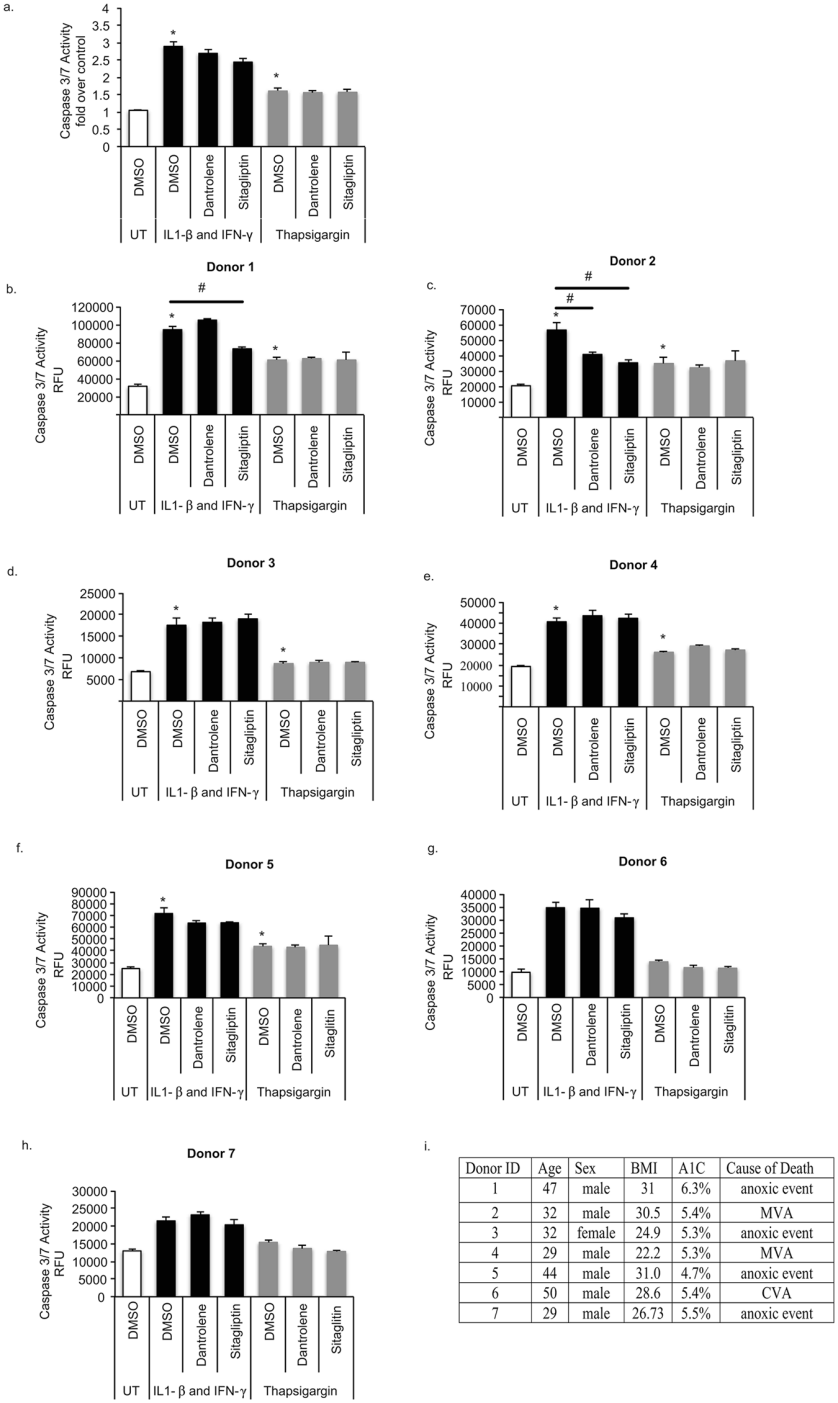
The Effect of Dantrolene and Sitagliptin on Cytokine Mediated Cell Death In Human Islets. (**a**–**h**) Human islet cells from 7 different organ donors were pre-treated for 24 hours with dantrolene 10 μM or sitagliptin 200 nM then stressed with cytokines (IL-1β 50 u/mL and IFN-γ 1000 u/mL) or thapsigargin 2 μM for 48 hours. Data are presented as combined results (panel a) as well as individual results (panel b–h) for all donors. Detailed information obtained from islet distribution centers on patient age, body mass index (BMI), hemoglobin A1C (A1C), Glucose Stimulated Insulin Release Stimulation Index (SI) and cause of death are presented in panel i. Data are expressed as mean ± SEM for all figures. ^#^p < 0.05 compared to cytokine or TG treated cells unpaired t-test.

To determine if the effects seen in a rodent cell line are translatable to humans, we next pre-treated human primary islets from seven different donors with dantrolene or sitagliptin prior to cytokine or thapsigargin treatment and monitored cell death (Fig. 7a–h). While certain islet batches showed that the drugs had a protective effect (Fig. 7b,c), overall the drugs did not have a statistically significant effect on cytokine- or thapsigargin-mediated caspase 3/7 activation (Fig. 7a).

## Discussion

**Figure 8:**
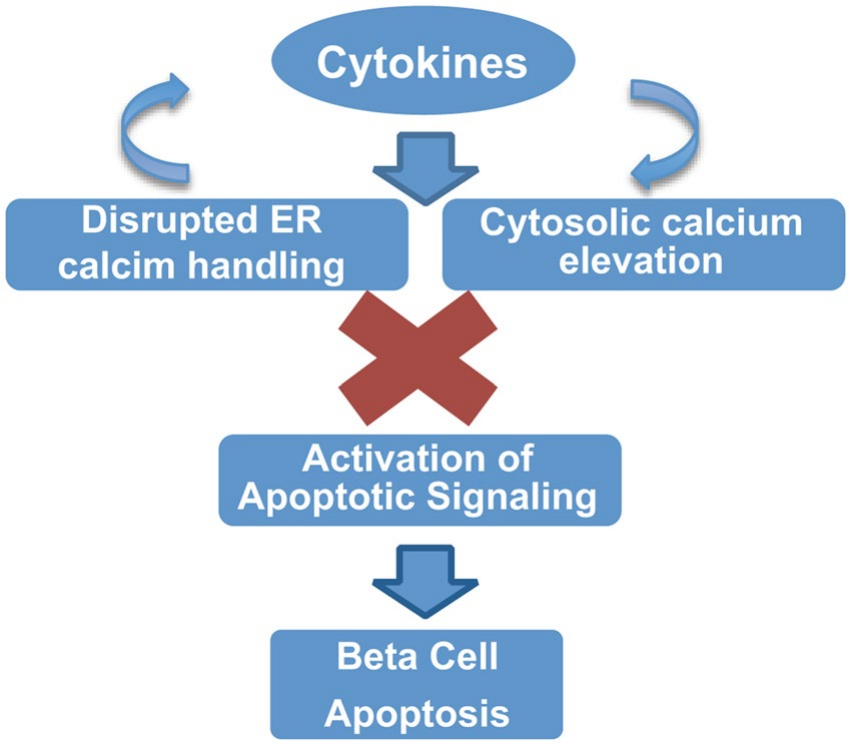
Cytokines Lead to Altered Cellular Calcium Homeostasis, and Induction of Βeta Cell Death. In beta cells cytokines cause increased ER stress, depletion of ER calcium, prevention of functional ER calcium release and elevation of cytosolic free calcium levels. This chain of events ultimately culminates in cell death. This study has proved that drugs targeting modulation of ER and cytosolic free calcium levels can prevent the activation of cytokine and ER stresses mediated pro-apoptotic pathways and decrease beta cell death.

In this study, we show that dantrolene and sitagliptin improve cytokine and ER stress-induced disruption of functional ER calcium release, partly prevent activation of the calcium-dependent protease calpain, and mitigate cytokine- and ER stress-mediated apoptosis in INS-1E cells. In addition, we have shown that TXNIP is a key mediator of cytokine-induced beta cell death. These results support stabilization of cellular calcium levels and TXNIP suppression as viable targets to protect against cytokine-mediated beta cell death in T1DM (Fig. 8).

The results of our human islet studies did not show a significant protection against cell death. While there is no *in vitro* system that perfectly mimics the pathophysiologic events in T1DM, human islets are often used to establish the translatability of a therapy. However, the fragility of primary human islets in culture along with the genetic and environmental heterogeneity of the donors often confound the translatability of human islet studies. Further studies utilizing various doses of dantrolene and sitagliptin may have yielded different results. However, due to the limited availablity of islets, this initial study focused on a single drug dose that showed effect in the first two donors. In additon, another commonly used model of T1DM, the Non-Obese Diabetic (NOD) mouse, may yield more results into the translatability of the drug effects into an *in vivo* model.

Although both dantrolene and sitagliptin partly decreased cytokine-mediated beta cell death in INS-1 E cells, we could not see complete protection of these cells from cytokine-mediated cell death. This is probably due to the fact that cytokine induces beta cell death through multiple mechanisms including ER calcium-depletion and oxidative stress^20, 38, 39^. Combination therapy of anti-oxidant and dantrolene or sitagliptin could be a viable option for suppressing cytokine-mediated beta cell death.

There are multiple potential therapeutic avenues to increase ER calcium levels. Increased levels of SERCA2b are protective against cytokines and ER stress-induced ER calcium depletion and beta cell death^16, 17, 20, 23^. Thus, targeting drugs that increase SERCA levels may be one way to prevent beta cell death in T1DM. Blockade of ER calcium release via the ryanodine and IP3 receptors may also prevent cytokine-mediated beta cell death. Ryanodine receptors are present in the ER membrane and are the major source of ER calcium efflux^40^. Blockade of the ryanodine receptor by the drug dantrolene has previously been shown to decrease ER stress-induced ER calcium depletion and beta cell death, and in the current study prevents cytokine-mediated beta cell death^25^. One concern with blocking ER calcium release is that insulin secretion is calcium dependent. However, previous work suggests that ryanodine receptor blockade does not negatively affect insulin release^41^. In addition, we have shown that dantrolene and sitagliptin have no effect on glucose stimulated insulin secretion in INS-1E cells and human islets. Thus, this study provides evidence that the blockade of the ryanodine receptors with drugs such as dantrolene or other novel compounds can decrease cytokine mediated beta cell death and may present a novel treatment for diabetes.

Pharmacologic modulation of cytosolic free calcium levels presents another pathway to prevent cytokine-induced beta cell death. Many beta cell stressors, including cytokines, hyperglycemia, and ER stress increase cytosolic free calcium levels^20^. Extreme or prolonged elevation of cytosolic free calcium induces activation of proteins and signaling cascades that result in beta cell death such as calpain^42^. Calpain hyper-activation has been shown to contribute to cell death in various diseases including diabetes^30, 31^. Beta cells have several isoforms of calpain and isoform 1,2 and 10 have specifically been associated with type 2 diabetes. However, no specific isoform has not been identified in association with T1DM^43–45^. This study demonstrates that inflammatory cytokines and ER stress induce calpain activation in INS-1E cells, which can be significantly diminished by treatment with dantrolene and sitagliptin. However, we also showed that suppression of calpain 1 and 2 activation alone is not sufficient to protect from cytokine mediated beta cell death. These data suggest that dantrolene and sitagliptin are likely affecting multiple pathways in addition to calpain activation to prevent cell death.

In this study we also identified that TXNIP expression was suppressed by sitagliptin. TXNIP is a pro-apoptotic component of the ER stress response and has been shown to be a critical link between ER stress and inflammasome activation^35, 36^. Verapamil and other calcium lowering agents have been shown to decrease levels of TXNIP^37^. Prior studies have also shown that verapamil can prevent hyperglycemia, obesity and low dose streptozotocin (STZ) related beta cell death^37^. Our study shows that sitagliptin decreases expression of TXNIP and that TXNIP suppression can completely prevent cytokine mediated caspase 3/7 activation. This suggests TXNIP may be a potent target to prevent beta cell death in T1DM.

Taken together, our studies support the hypothesis that drugs targeting stabilization of cellular calcium levels and suppression of TXNIP during pro-inflammatory cytokine stress can prevent beta cell death, thus establishing modulation of ER and cytosolic free calcium levels and TXNIP suppression as viable options to prevent cytokine-mediated beta cell loss in diabetes.

## Methods

### Reagents

Thapsigargin, pioglitazone, dantrolene, verapamil and calpain inhibitor III were purchased from Sigma. Sitagliptin was purchased from Biovision Inc. Anti-GAPDH, anti-CHOP, anti-calpain 2 and anti-tubulin antibodies were purchased from Cell Signaling. Anti-fodrin antibody for cleaved spectrin measurements was purchased from Enzo Life Sciences. Anti-SERCA antibody was purchased from Santa Cruz Biotechnology. Anti-TXNIP antibody was purchased from MBL International. 1:1000 dilution was used for immunoblot. Rat and human IL1-β and IFN-γ were purchased from R&D systems. Fluo-4 AM was purchased from Invitrogen/Life Technologies. SiRNA directed against CAPN2 and control scramble siRNA were obtained from Origene Technologies.

### Cell culture

INS-1E cells were a kind gift of Prof. Pierre Maechler, University of Geneva and not cotaminated with mycoplasma. INS-1E cells were cultured in RPMI-1640 medium supplemented with 10% FBS, antibiotics, sodium pyruvate, glutamax, non-essential amino acids, HEPES, and β-mercaptoethanol. Human Embryonic kidney (HEK)-293 cells used in lentiviral production were cultured in DMEM containing 10% FBS and antibiotics. To establish INS-1E cell lines stably expressing the D1ER calcium probe, cells were transduced with lentivirus expressing D1ER and selected by cell sorting via FACS. The calcium-sensing D1ER plasmid was generously gifted from Dr. Amy Palmer (University of Colorado, Boulder, Colorado) and subcloned into a lentiviral plasmid. SiRNA transfections were performed using TransIT (Mirus Bio LLC) per the manufacturer’s protocol.

### Human Islet Studies

De-identified human islet preparations were acquired from PRODO labs or the Integrated Islet Distribution Program (IIDP). Informed consent was obtained from all subjects. Experiments were approved by Washington University’s Human Research Protection Office and conducted in accordance with the National Institutes of Health guidelines. Islets were allowed to recover overnight prior to experiments and were cultured in CMRL medium supplemented with FBS and penicillin/streptomycin. Islets were dispersed with 0.05% trypsin EDTA and then plated to a 96 well plate for experiments.

### Measurement of ER calcium levels

INS-1E cells expressing D1ER cameleon were plated onto 6-well plates, treated with each compound for the indicated times, and then harvested by trypsinization. After washing with PBS, cells were resuspended in the Hanks’ buffered salt solution. Flow cytometry analyses were performed with LSRII (BD) at the FACS core facility of Washington University School of Medicine. The results were analyzed by FlowJo version 7.6.3 (Tree Star).

### Measurement of apoptosis

INS-1E cells were plated to 96 well plates and were pre-treated with drugs or drug combinations for 24 hours. After pre-treatment, cytokine cocktail (IL1-β 50 ng/mL, IFN-y 50 ng/mL) or thapsigargin 10 nM was added to the cells for 24 hours. Caspase 3/7 activity was then detected using the Caspase-Glo® 3/7 Assay (Promega) using the Infinite M1000 plate reader (Tecan).

### Quantitative real-time PCR

Total RNA was extracted using RNeasy kits (QIAGEN). RTPCR was performed using ImPromII (Promega) reverse transcriptase, and quantitative PCR was performed using the Thermoscientific ViiA 7 real-time PCR system using SYBR green dye (Life Technologies). Primers were obtained from Integrated DNA Technologies (IDT) and sequences were as follows (sense and anti-sense); rat β-actin 5′-GCA AAT GCT TCT ACG CGG AC-3′ and 5′-AAG AAA GGG TGT AAA ACG CAG C-3′, Rat BIP 5′-TGG GTA CAT TTG ATC TGA CTG GA-3′ and 5′-CTC AAA GGT GAC TTC AAT CTG GG-3′, Rat CHOP 5′-CTC ATT CTC CTG CTC CTT CTC C-3′ and 5′-AGA GTG GTC AGT GCG CAG C-3′, Rat SERCA2b 5′-TTT GTG GCC CGA AAC TAC CT-3′ and 5′-GGC ATA ATG AGC AGC ACA AAG GG-3′.

### Measurement of SERCA Activity

The SERCA activity assay was performed as previously described^46^. INS-1E cells were lysed in a hypotonic buffer consisting of 10 mM NaHCO_3_, 250 mM sucrose, 5 mM NaN_3_, and 1 mM PMSF. ER fractions were enriched using differential centrifugation. 50–75 μg of ER protein fractions were added to the assay mixture 100 mM KCL, 30 mM imidazole-histidine (pH 6.8), 5 mM MgCl_2_, 5 mM ATP, 5 mM (COOK)_2_, 5 mM NaN_3_ and 50 μM CaCl_2_ (10 μCi/μmol [^45^Ca]; CaCl_2_ American Radiolabled Chemicals) which was heated to 37 degrees for 15 minutes. The reaction was stopped by the addition of 250 mM KCl and 1 mM LaCl_3_. The mix was then vacuum filtered through a 0.2 um HT Tuffryn membrane (Pall). SERCA-dependent calcium transport was measured by using the SERCA inhibitor, 10 μM thapsigargin.

### Measurement of cytosolic free calcium activity

Cytosolic free calcium activity was assayed in INS-1E cells pre-treated for 24 hours with dantrolene 100 nM, sitagliptin 200 nM or drug combination followed by exposure to cytokine cocktail (IL1-β 50 ng/mL, IFN-y 50 ng/mL), or thapsigargin 10 nM. Cytosolic free calcium activity was measured via the Fura-2 No Wash Calcium Assay (Abcam) per the manufacture’s protocol. Briefly, cells were plated in 96-well plates and stained with Fura-2 AM per manufacturers protocol. Fluorescence was measured at excitation wavelengths at 340 nm and 380 nm and emission wavelength at 510 nm. Then background was subtracted for both excitation wavelengths and the resulting values were used to calculate 340/380 ratios.

### Measurement of caffeine stimulated calcium release

Confocal microscopy experiments were performed on a Zeiss LSM 880 using a C-Apochromat 40x Water NA 1.2 objective lens. Cells were pretreated with dantrolene 100 nM or sitagliptin 200 nM then exposed to the cocktail of cytokines (IL1-β 50 ng/mL, IFN-y 50 ng/mL). The cell permeable calcium sensor Fluo-4 AM (Invitrogen) was then added at a concentration of 4 µM in 1 ml of RPMI or PBS for 15–20 minutes. Cells were washed to remove excess Fluo-4AM and drugs prior to imaging. Experiments were performed in 37 °C RPMI or 37 °C calcium-free DPBS. The incubator stage provided the physiological conditions of 37 °C and 5% CO_2_. Images were acquired as a time series of 2 minutes for each condition before and after the addition of 5 mM caffeine. Since Fluo-4 AM is a single-wavelength calcium sensor, the data shown are related to the variation of fluorescence intensity of the stimulated cells (ΔF) relative to the basal condition (F_0_). Signals from more than 50 cells were averaged for each condition to avoid differences due to different Fluo-4 concentrations in the cells. Photostability experiments were performed prior to each imaging session to verify the best acquisition conditions to give minimal photobleaching. Data analysis and simple image processing were carried out with ImageJ.

### Measurement of glucose mediated insulin secretion

INS-1E cells or human islets were treated with dantrolene and sitagliptin for 24 hours. In addition dantrolene and sitagliptin were kept in KRB buffer during GSIS. INS-1E cells were incubated in KRB buffer with 0 mM glucose for 40 minutes at 37 degrees. Cells were then incubated with KRB buffer and 2.8 mM glucose for 1 hour followed by KRB with 16.7 mM glucose for 1 hour at 37 degrees. Supernatant was collected for insulin quantification at the end of each incubation. Insulin levels were quantified using the Rat/Mouse insulin ELISA kit from EMD Millipore. Cells were lysed at the end of the experiment and protein was quantified using a NanoDrop system from Thermo Scientific. Data was presented as total insulin level normalized to total protein.

Human islets from 3 different donors were hand picked and 8–10 islets were transferred to an eppendorf tube. Islets were incubated in KRB buffer with 3.3 mM glucose for 40 minutes. Islets were then exposed to either 3.3 mM glucose or 16.7 mM glucose KRB buffer and after one hour buffer was collected for insulin analysis. Insulin levels were measured using the Stellux human insulin ELISA kit from ALPCO. DNA was extracted from islets using the Qiagen DNeasy Blood and Tissue Kit per protocol. DNA was quantified using a NanoDrop system from Thermo Scientific. Data was presented as total insulin level normalized to total DNA.

### Statistics

All results are shown as mean ± SEM. Two-tailed Student’s t-tests were used to compare two treatments. One-way ANOVA was performed using Graph Pad Prism software 6.0 for multiple comparisons. Levels of statistical significance were indicated as follows; *p < 0.05 compared to control and ^#^p < 0.05 compared to cytokines or thapsigargin treated cells.

## Electronic supplementary material

Supplementary Figure 1
