# Supplementary material for: Targeting Cellular Calcium Homeostasis to Prevent Cytokine-Mediated Beta Cell Death
Source: Sci Rep. 2017 Jul 17;7:5611. doi: 10.1038/s41598-017-05935-4 (PMC5514111; doi:10.1038/s41598-017-05935-4)

Targeting Cellular Calcium Homeostasis to Prevent Cytokine-Mediated Beta Cell Death

Amy L. Clark[1], Kohsuke Kanekura[2], Zeno Lavagnino[3], Larry D. Spears[4], Damien Abreu[4], Jana Mahadevan[4], Takuya Yagi[4], Clay F. Semenkovich[3,4,] David W. Piston[3], and Fumihiko Urano[4,5 ¶]

[1]Department of Pediatrics, Washington University School of Medicine, St. Louis, MO 63110, USA;
[2]Department of Molecular Pathology, Tokyo Medical University, Tokyo 160-8402, Japan;
[3]Department of Cell Biology and Physiology, Washington University School of Medicine, St. Louis, MO 63110, USA;
[4]Department of Medicine, Division of Endocrinology, Metabolism, and Lipid Research, Washington University School of Medicine, St. Louis, MO 63110, USA;
[5]Department of Pathology and Immunology, Washington University School of Medicine, St. Louis, MO 63110, USA.

[¶]***Corresponding author:***
Fumihiko Urano, M.D., Ph.D.
Department of Medicine,
Washington University School of Medicine
Email: urano@wustl.edu

Supplementary Figure Legends

Supplementary Figure 1. Full-length blots for Figure 3a, Figure 4b, Figure 4e, and Figure 5a.

Figure 3a SERCA

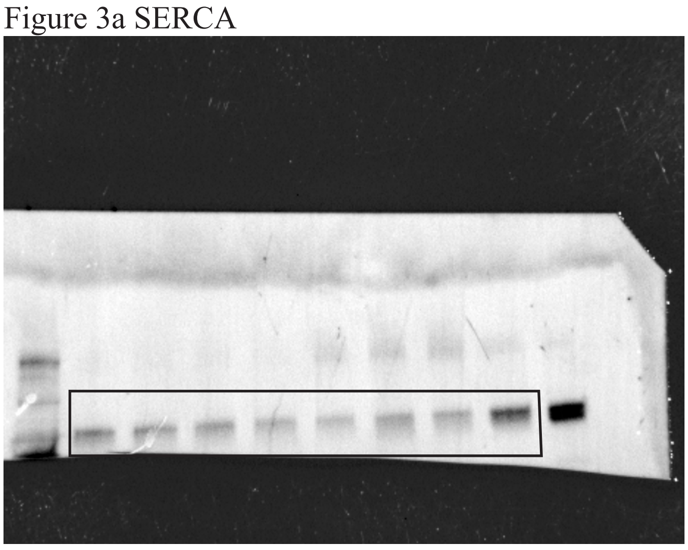

Figure 3a Tubulin

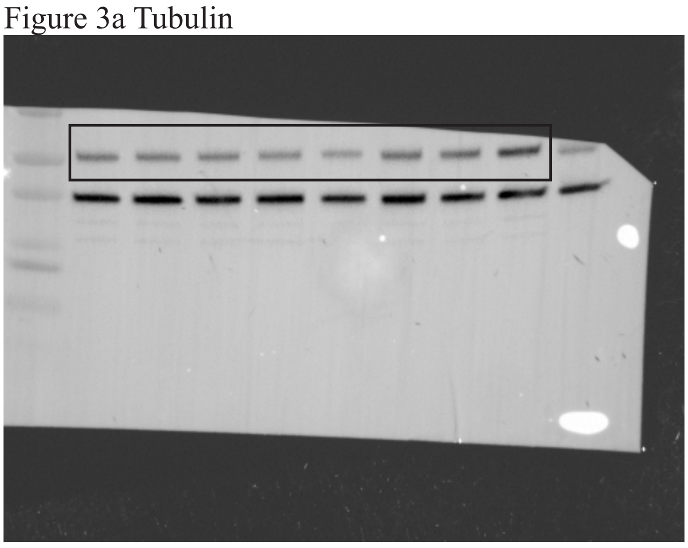

Figure 4b Spectrin Cytokines

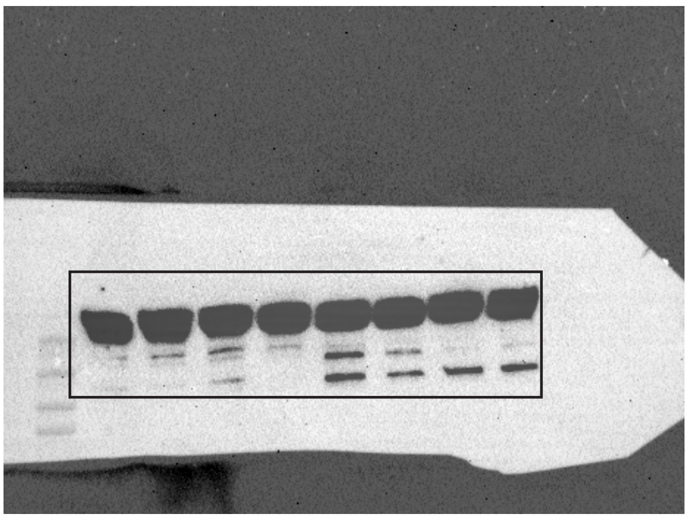

Figure 4b Tubulin and GAPDH Cytokines

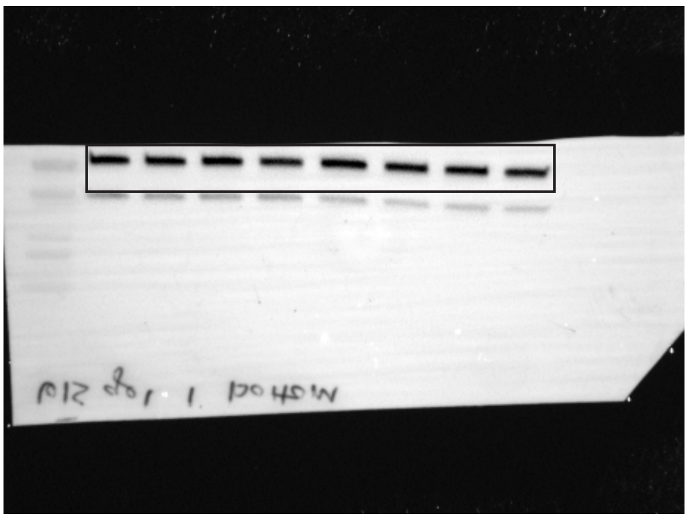

Figure 4b Spectrin Thapsigargin

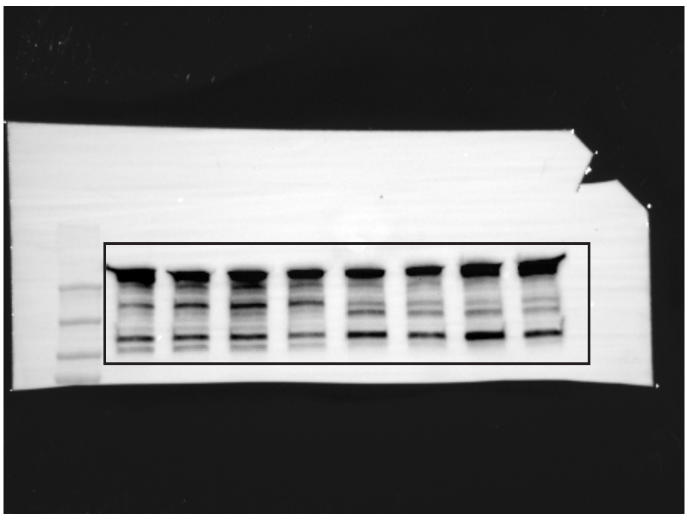

Figure 4b Tubulin Thapsigargin

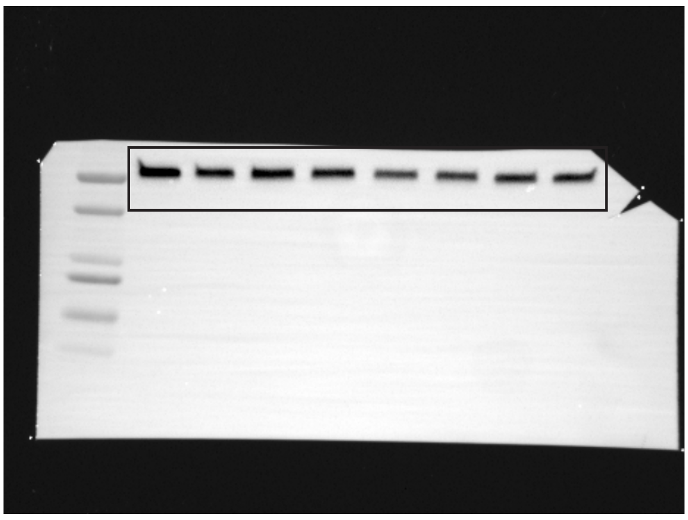

Figure 4e Calpain 2 and GAPDH

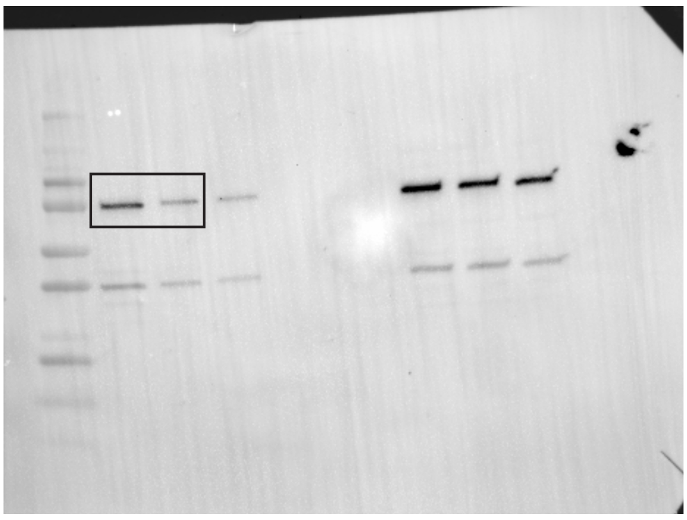

Figure 4e GAPDH

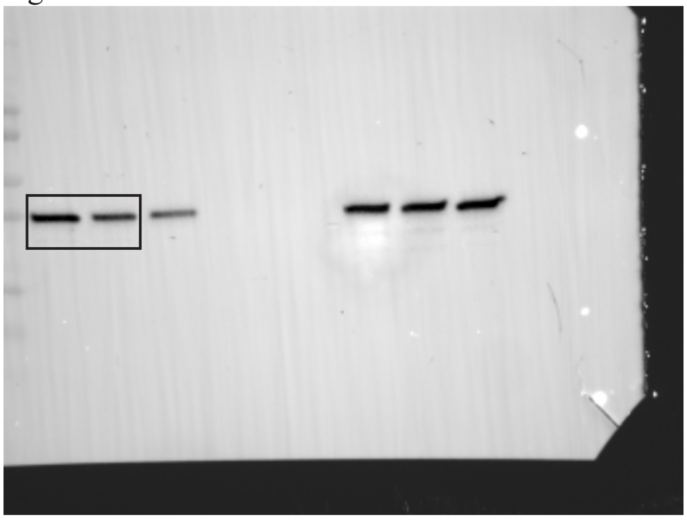

Figure 5a TXNIP and GAPDH high exposure

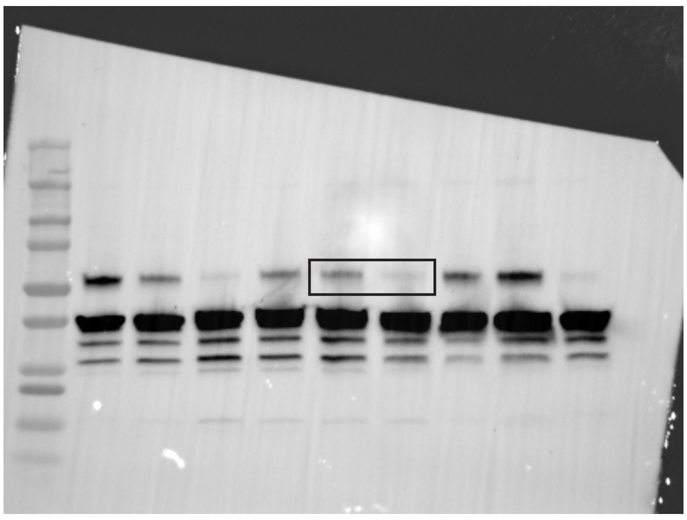

Figure 5a TXNIP and GAPDH low exposure

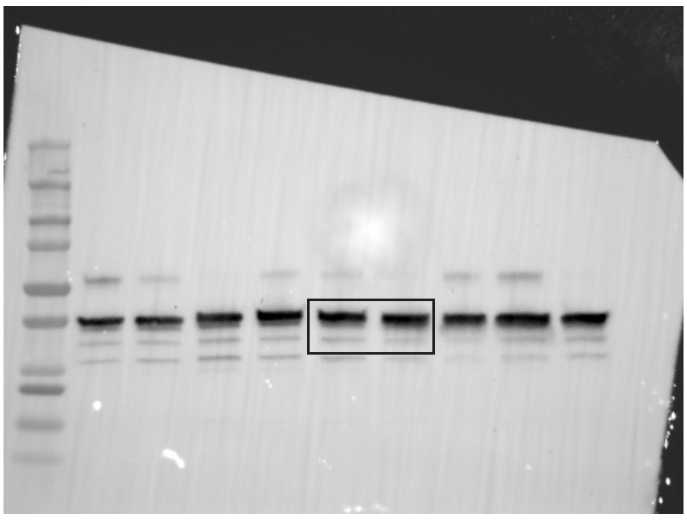

Supplement: Supplementary file 1 — Supplementary Figure 1 [file 41598_2017_5935_MOESM1_ESM.pdf]
